# Laryngo-tracheo-oesophageal clefts

**DOI:** 10.1186/1750-1172-6-81

**Published:** 2011-12-07

**Authors:** Nicolas Leboulanger, Eréa-Noël Garabédian

**Affiliations:** 1 Paediatric Otolaryngology - Head and Neck surgery department, UPMC - Paris VI University, Armand-Trousseau Children's Hospital, 26 avenue du Dr Netter, 75012 Paris - France

## Abstract

A laryngo-tracheo-esophageal cleft (LC) is a congenital malformation characterized by an abnormal, posterior, sagittal communication between the larynx and the pharynx, possibly extending downward between the trachea and the esophagus. The estimated annual incidence of LC is 1/10,000 to 1/20,000 live births, accounting for 0.2% to 1.5% of congenital malformations of the larynx. These incidence rates may however be underestimated due to difficulty in diagnosing minor forms and a high mortality rate in severe forms. A slightly higher incidence has been reported in boys than in girls. No specific geographic distribution has been found. Depending on the severity of the malformation, patients may present with stridor, hoarse cry, swallowing difficulties, aspirations, cough, dyspnea and cyanosis through to early respiratory distress. Five types of laryngo-tracheo-esophageal cleft have been described based on the downward extension of the cleft, which typically correlates with the severity of symptoms: Type 0 laryngo-tracheo-esophageal cleft to Type 4 laryngo-tracheo-esophageal cleft. LC is often associated with other congenital abnormalities/anomalies (16% to 68%), mainly involving the gastro-intestinal tract, which include laryngomalacia, tracheo-bronchial dyskinesia, tracheo-bronchomalacia (mostly in types 3 and 4), and gastro-esophageal reflux disease (GERD). The syndromes most frequently associated with an LC are Opitz/BBB syndrome, Pallister Hall syndrome, VACTERL/VATER association, and CHARGE syndrome. Laryngeal clefts result from failure of fusion of the posterior cricoid lamina and abnormal development of the tracheo-esophageal septum. The causes of the embryological developmental anomalies leading to LC are not known but are thought to be multifactorial. LC appears to be mostly sporadic although some familial cases with suspected autosomal dominant transmission have been reported. The age of diagnosis depends mainly on the severity of the clinical symptoms and therefore on the extent of the LC. Diagnosis is made either based on clinical manifestations or on investigations, such as endoscopy, X-ray, CT scan, performed for other conditions. Differential diagnoses include tracheo-bronchial fistula, gastro-esophageal reflux disease and neurological swallowing disorders, as well as laryngomalacia and laryngeal palsy. Prenatal diagnosis of LC has never been reported, although associated anomalies may be detected on fetal ultrasonography. Once the cleft is diagnosed, it is essential to determine its length to orient the management and treatment approach. Management involves maintenance of satisfactory ventilation, prevention of secondary pulmonary complications as a result of repeated aspirations, and adequate feeding. Endotracheal intubation may be required for respiratory distress in severe cases. Treatment requires endoscopic or external surgery to close the cleft. Surgery should be performed as early as possible to avoid complications related to aspiration and gastric reflux, except in type 0 and type 1 cases in which conservative measures must first be attempted. The prognosis is variable depending on the severity of the LC and associated malformations. Early diagnosis and appropriate treatment and management help to reduce mortality and morbidity.

## Review

### Historical delineation and disease definition

The first reported clinical case of laryngo-tracheo-oesophageal cleft was made by Richter in 1792 in a newborn presenting with recurrent aspiration [1]. The first successful surgical reconstruction was performed in 1955 [2], and the first reliable classification system was proposed in 1965 after a review of all available literature [3]. The first large and well documented series, illustrating the guidelines of diagnostic and therapeutic strategies, was proposed in 1983 [4]. Since then, numerous manuscripts have been published regarding laryngo-tracheo-oesophageal clefts and their management.

**Figure 1:**
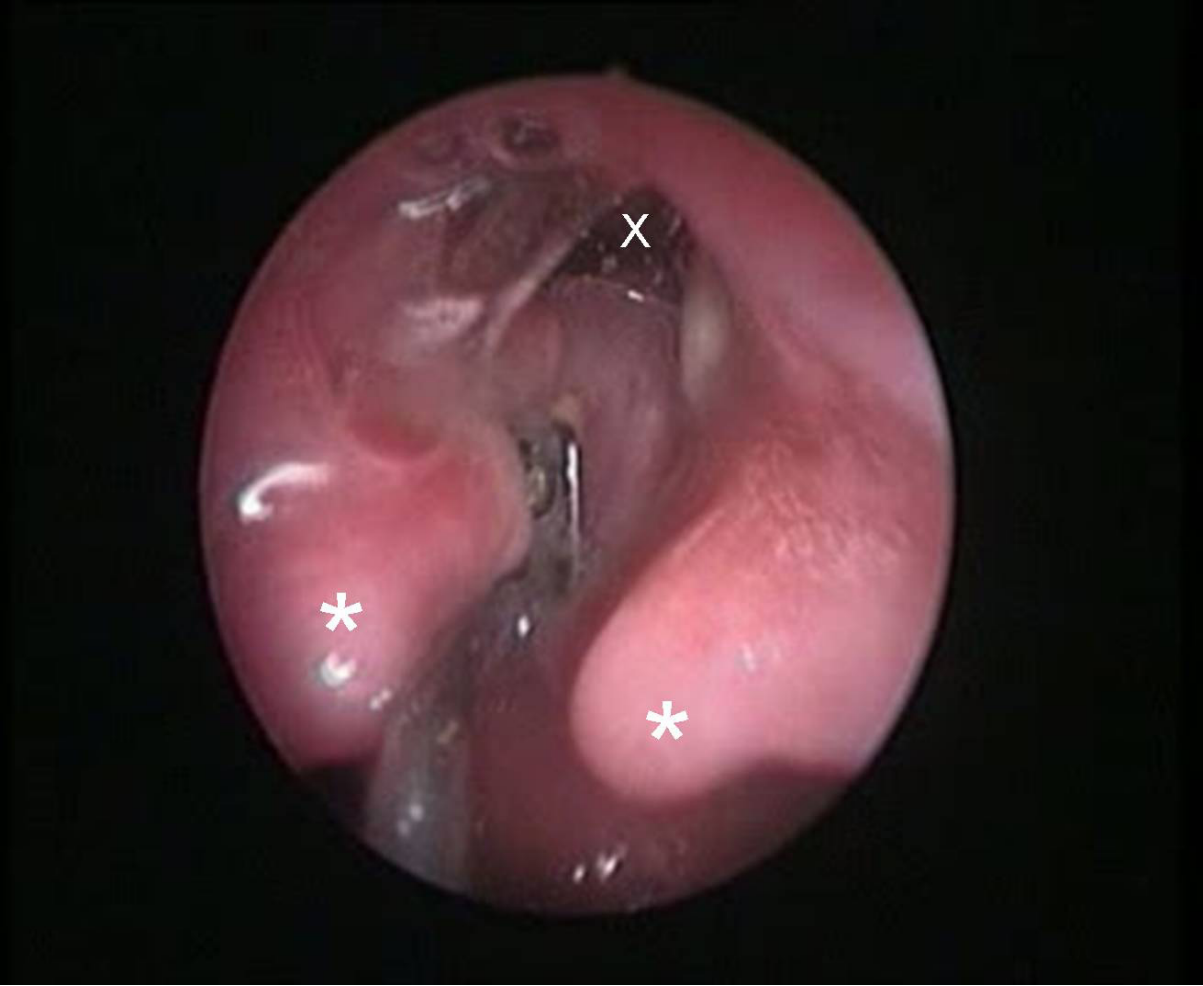
**Type III LC, endoscopic view**. *: arytenoids; X: tracheal lumen. Palpation of the cleft.

. A laryngeal-tracheo-oesophageal cleft (commonly termed laryngeal cleft, LC) is a congenital malformation of the posterior part of the larynx, possibly extended to the trachea, creating an abnormal communication between the laryngo-tracheal axis and the pharyngo-oesophageal axis (Figure 1). Thus, the physiological separation between the airway and the digestive tract is lost, leading to chronic cough, aspiration, respiratory distress, pneumonia.... The severity of a LC is directly correlated to the downward extension of the cleft. This disease is registered in both Orphanet (ORPHA2004) and OMIM (#215800) databases [5].

Recent advances in knowledge, diagnosis and, above all, the treatment of LC, have led to significant improvements in survival and quality of life of these patients.

### Classifications

Over the past 50 years, many classifications of LC have been proposed, all based on the downward extension of the cleft: Petterson (1955), Armitage (1984), Evans (1985), Benjamin (1989), Meyer (1990), DuBois (1990), and Sandu (2006) [6]. All have a threefold interest:

- Descriptive; allowing the comparison of a series of patients in the literature;

- Therapeutic; influencing the choice of a reconstructive technique and surgical approach

- Prognostic; as success and survival rates are highly correlated to the extension of the LC.

**Figure 2:**
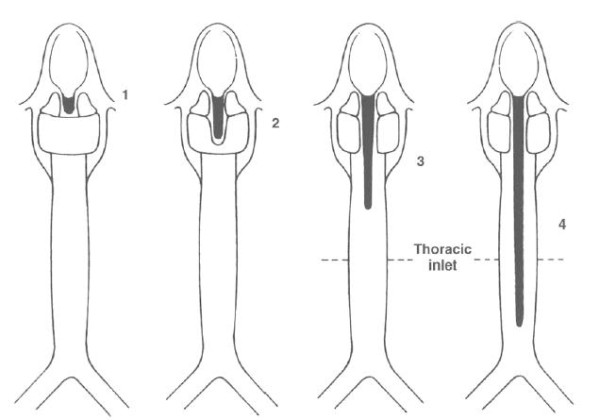
**Benjamin and Inglis' original classification **[7] .

To date, the Benjamin and Inglis classification [7], modified by Sandu in 2006 [8], are the most frequently used (Figure 2). Indeed, they differentiate partial and total cleft of the cricoid cartilage, as well as cervical and tracheo-thoracic cleft. Those elements are essential in the choice of a therapeutic strategy.

Type 0: submucosal cleft

Type I: supraglottic, interarytenoid cleft, above the vocal fold level

Type II: cleft extending below the vocal folds into the cricoid cartilage

Type III a: cleft extending through the cricoid cartilage but not into the trachea

Type III b: cleft extending through the cricoid cartilage and into the cervical trachea

Type IV: cleft extending into the thoracic trachea, potentially down to the carina

Submucosal LC was initially described by Tucker in 1987 [9] as a posterior, sagital, submucosal, cartilage defect with intact soft tissues (mucosa and interarytenoid muscle). It is clinically relevant, since it has been described with other anomalies of the cricoid cartilage, including congenital subglottic stenosis [10].

### Epidemiology

LC is a rare, congenital, anomaly. It is widely accepted that its incidence is probably underestimated. Indeed, 1) minor LC (type 0 or type 1) may either be asymptomatic or show only mild symptoms; 2) the endoscopic diagnosis is difficult and several reports exist a missed malformation despite a well-conducted endoscopic assessment; 3) high-grade LC (type 4) have a high mortality rate, often leading to the patient's death before diagnosis can be made; 4) the endoscopic assessment may not be a priority in cases with numerous associated malformations.

As such, the estimated incidence of LC is 1 in 10,000 to 20,000 living births [6,11,12], representing about 0.2% to 1.5% of the congenital malformations of the larynx [10,13-17].

From 1990 to 1995, of the 2,338 endoscopies carried out in a specialized paediatric department, only 7 cases of laryngeal cleft were identified (0.3%) [10]. In 1971, a review of 433 cases of laryngeal malformations identified 2 cases of LC (0.5%) [17]. An initial study conducted between 2002 and 2005, found among 264 patients evaluated for chronic cough or aspiration, 20 cases of type I LC (7.6%) [18]. This incidence, higher than that previously mentioned, appears to be more in line with reality due to: 1) a better understanding of the disease and its diagnosis and 2) a pre-selection of patients.

LC has a slightly higher incidence in boys than in girls, with a ratio of 1.2 [19] to 1.8 [20]. Even if data on this subject is scarce, no evidence appears to exist relating to an occurrence of a specific racial predominance [20]. Cases of LC appear to be mostly sporadic. However, reports exist of families with multiple children having a cleft. In these patients, the possibility of an autosomal dominant transmission has been suggested [21]. Alcohol and/or drug abuse during pregnancy, multiple miscarriages, hydramnios and prematurity are frequently reported, but none have been proven to be a specific risk factor [1,10].

### Etiopathology: embryology and animal model

In the classic embryological description of the respiratory system, the larynx develops from two tissues evolving in parallel: the endoderm coming from the foregut and the mesenchymal elements from the 4^th ^and 6^th ^branchial arches [22]. The division of the foregut is the result of the fusion of lateral ridges appearing in the lateral walls of the foregut. This process starts caudally and ends cranially to the region of the larynx, thus forming a septum that divides the foregut into a ventral portion - the laryngo-tracheal tube - and a dorsal portion - the oesophagus.

Recently, researchers studying the embryology of the foregut of chick embryos, failed to identify subtle lateral ridges and suggested that the development of both the trachea and the oesophagus could result from a size reduction of the foregut, caused by a system of folds that get close but do not merge [22]. This system of folds could appear in the foregut and involve the tracheo oesophageal space in both the cranial and caudal areas: the caudal with a cranial development and the two cranial folds with a caudal evolution. Moreover, the respiratory diverticulum has also been described; the latter developing from the ventral area of the foregut and continuing to elongate downward to form the trachea [23]. The portion of the mesenchyme, known as the tracheo-oesophageal septum, located between the digestive and respiratory tracts, is the result of the separation of the two tracts. Apoptotic mechanisms are also involved in the growth and evolution of tissues: apoptotic epithelial cells can be found at the tracheo-oesophageal separation site in the ventral part of the foregut, where cellular activity is intense, whereas the dorsal part remains inactive [24].

Several models have been proposed to explain tracheo-oesophageal anomalies, including LC [25]:

- *Intra-embryonic pressure*: an intense curvature of the cervical region, during the heart's development, could cause a strain and a displacement of the oesophagus, resulting in growth anomalies.

- *Epithelial occlusion*: the oesophagus has a solid stage of development, and eventually re-canalizes. A failure of this latter process could result in malformations.

- *Vascular occlusion*: the persistence of an abnormal vessel could lead to vascular insufficiency in the foregut, resulting in growth anomalies.

- *Differential cell growth*: abnormal cell development in either the ventral or dorsal part of the developing tracheo-oesophagus could result in oesophageal or tracheal defects.

It has been suggested that a premature arrest in development of the tracheo-oesophageal septum, and the lack of fusion of the two lateral parts of the developing cricoid cartilage, could be responsible for LC [3]. This model, however, does not explain the mechanism of associated malformation, such as laryngeal atresia and tracheo-oesophageal fistula.

An animal model of laryngo-tracheal malformations has been described in rat embryos exposed to *doxorubicin *(Adriamycin^®^) [25-28]. After exposure, these embryos display major tracheo-oesophageal anomalies, similar to those described in the VACTERL association (oesophageal atresia and tracheo-oesophageal fistula). However, LC has not yet been observed in this model under experimental conditions.

### Clinical description

#### Clinical symptoms

The intensity of clinical symptoms of laryngeal clefts typically correlates with the type of cleft itself [10,19]. Most frequent symptoms are summarized in Table 1.

**Table 1:** Most frequent clinical symptoms [1]

Swallowing disorders (50%)	Aspiration and cyanosis during feeding (53 to 80%) Chronic cough (27 to 35%)
Pharynx and larynx (43%)	Stridor (10 to 60%) Toneless or weak cry (16%) Pharyngeal hypersecretions (10 to 23%)

Respiratory (37%)	Recurrent pneumonia (16 to 54%) Respiratory distress at birth

**Type 0 **clefts may display mild to no obvious symptoms (occasional aspiration) when clinically isolated, but association with other airway malformations or syndromes is possible. They are often discovered during an endoscopy or an external procedure initiated for other reasons, and their diagnosis is especially difficult if the surgeon is not aware of their clinical appearance [9,29,30].

**Type I **clefts usually present with mild to moderate symptoms, including stridor, a toneless or hoarse cry, and swallowing disorders. Aspiration, cough, dyspnoea and cyanosis during feeding are possible but not routine. The impact on the pulmonary tract is usually none to mild.

**Type II and III **clefts usually display more swallowing disorders (aspiration mostly) and pulmonary tract infections.

**Type IV **has a poor prognosis, due to the early respiratory distress they cause, and to the difficulty of maintaining correct mechanical ventilation [20,31-33].

However, some cases of large LCs with a surprisingly little symptomatology have been documented. It is supposed that the excess of oesophageal mucosa herniating into the cleft in the LC provides, in these cases, some degree of protection against aspiration. However, this mucosal hernia has also been proposed as the cause of stridor and airway obstruction.

The age at which a diagnosis is made depends mostly on the severity of the clinical symptoms, and therefore on the extent of the LC itself. Moreover, the experience of the medical team managing the LC may also influence the age at which a diagnosis is made, especially when the associated malformations are mild or none [14,16,34]. In the literature, the age at diagnosis is very variable. Thus, in one series, type 0 LC were diagnosed at an average age of 6 months, type I before the age of 6 months and type II before the age of 2 months [19]. In another series, [29], the average age at diagnosis, regardless of the type of cleft, was 15 days to 12 years of age.

Thus, every swallowing disorder (cough during feeding, aspiration and/or cyanosis) should lead to an endoscopic examination of the child's airways to assess for a LC.

#### Associated syndromes

LC is often associated with other congenital abnormalities (16% to 68%), mostly malformations of the digestive tract [4,29]. A full examination of both digestive and respiratory tracts is therefore mandatory during the assessment of a LC.

These associated malformations may be syndromic or appear isolated. Four syndromes are among the most frequently associated with a LC:

- The Opitz G/BBB syndrome (Orpha2745/OMIM #145410), characterized by laryngeal malformations (including LCs) which are associated with craniofacial anomalies (pinna malformations, cleft lip and palate, hypertelorism), genitourinary anomalies (hypospadias), and other malformations of the ventral midline. Two forms exist: one with an autosomal dominant inheritance and the other with an X-linked inheritance.

- The 22q11 monosomy (CATCH 22, 22q11 microdeletion, DiGeorge syndrome...) (Orpha567/OMIM #192430 and #188400). This syndrome may include numerous malformations especially hypoplasic thymus and parathyroid glands, cardiopathy, velopharyngeal insufficiency with or without cleft palate, and sometimes LC. The clinical course mainly depends on the malformations involved. Its overall incidence is estimated at 1/5000 births, but is much lower when associated with LC.

- The Pallister Hall syndrome (Orpha672/OMIM #146510) is characterized by the association of laryngeal (LC), gastrointestinal, cardiopulmonary, limb (polydactyl and syndactyl) and neurological malformations (congenital hypothalamic hamartoblastoma with hypopituitarism). Its inheritance is autosomal dominant.

- The VACTERL association (Orpha887/OMIM #192350). Its aetiology is still unknown in man. The acronym stands for Vertebral, Anal, Cardiac, Tracheo-oesophageal (including LC), Ear (middle and inner), Renal, and Limb malformations.

- The CHARGE syndrome (Orpha138/OMIM #214800) results in the main, from a CHD7 gene mutation. The acronym stands for Coloboma, Heart malformations, choanal Atresia (uni- or bilateral), growth and mental Retardation, Genitourinary malformations, and Ear malformations (external, middle, and inner ear). Several other malformations may also be associated, some of them constant (e.g. olfactory bulb hypoplasia and semi-circular canal anomalies). An LC may also be associated with many other cranial malformations (cleft lip and palate, laryngomalacia, laryngeal webs...).

Furthermore, several other associated malformations are possible as shown in Table 2. It is likely that most of these malformations are in fact linked to not-yet identified syndromes. However, a purely isolated LC is also possible.

**Table 2:** Frequent non-syndromic associated malformations [1]

Localisation	Malformation
Digestive tract (16 to 67%) [19,31,32,45,46]	Esophageal atresia (20 to 37%) Tracheo-esophageal fistula (10 to 20%) Anal atresia (21%) Abnormal intestinal rotation (13%) Meconium ileus (8%) Microgastria Exomphalos

Genitourinary tract (14 to 44%) [39]	Hypospadias (7 to 13%) Kidney malformation (4%) Inguinal hernia, cryptorchidism

Cardiovascular system (16 to 33%)	Coarctation of the aorta, transposition of the great vessels Ductus arteriosus

Craniofacial (5 to 15%)	Cleft lip and palate (5%) Choanal atresia Micrognathia, glossoptosis Hypertelorism, dysmorphia Anomaly of the external ear

Tracheo-broncho-pulmonary (2 to 9%) [46]	Short trachea Bronchial, tracheal stenosis Abnormal lung segmentation, hypoplasia

### Diagnostic methodology

#### Endoscopic examination

Several different diagnoses are possible, and among them tracheobronchial fistulas, laryngomalacia, laryngeal palsy, gastro-oesophageal reflux (GERD), neurological swallowing disorders... Most of these diagnoses can be ruled out during the endoscopic examination.

The endoscopic assessment is essential to LC diagnosis. It must be conducted with special care and attention, because it is possible to miss a low-grade malformation if the examiner is not especially aware of it [15].

Such endoscopic examination must be conducted in the operating room and includes:

- A fiberoptic examination of the larynx under local anaesthesia, looking for laryngomalacia, direct aspiration, and assessing laryngeal mobility and sensitivity. A LC may be suspected at this time, but usually cannot be diagnosed with certainty [29].

- A full examination of the airways and the oesophagus with rigid telescopes, under general anaesthesia with spontaneous breathing, in order not to miss a tracheo-bronchial dyskinesia, or a tracheo-bronchial laryngomalacia (50 to 66%). The entire respiratory tract must be assessed because tracheo-bronchial fistulas are associated in 10 to 60% of the cases [34,35].

**Figure 3:**
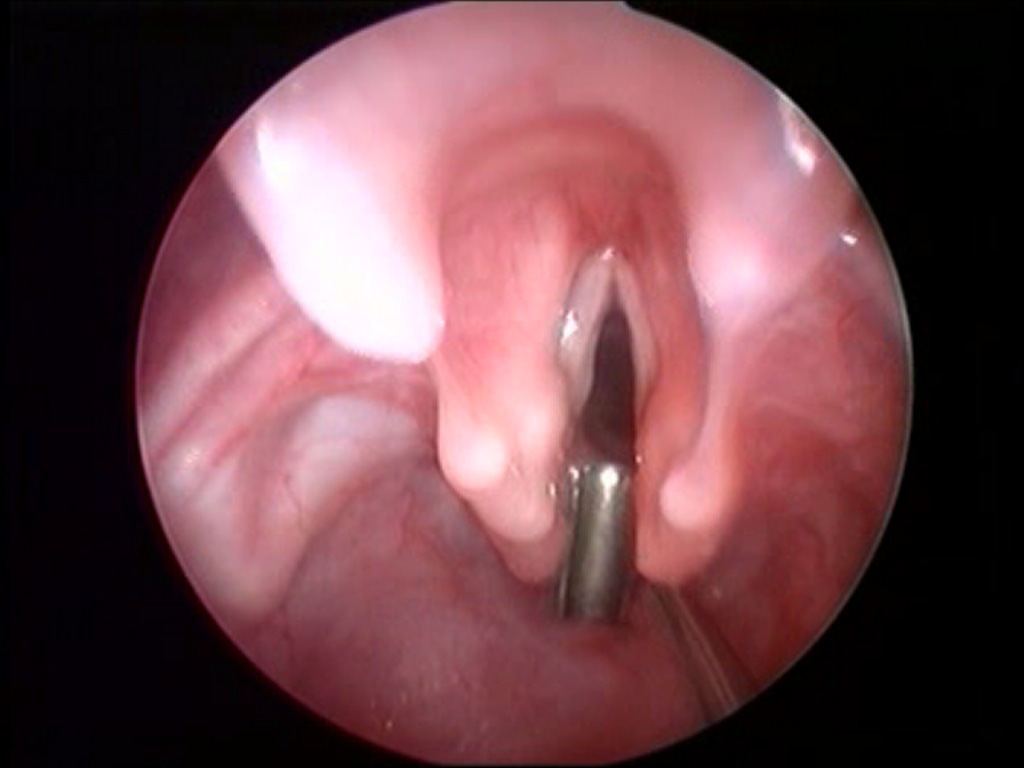
**Endoscopic view; palpation of the posterior cleft**.

The larynx must then be examined with microlaryngoscopy during a laryngeal suspension, using high magnification either with a microscope or video monitored telescope [36]. A preliminary topical application of 1% *lidocaine *may help before cleft palpation. The posterior glottis must be carefully inspected and palpated, looking for a sagittal cleft between the digestive and the respiratory tracts (Figure 3). A LC may be inadvertently overlooked because of the redundant mucosa between the oesophagus and trachea, prolapsing into the cleft. Several probes have been designed and proposed for cleft palpation [16]. Once the diagnosis is made, it is fundamental to assess the length of the cleft [11].

The endoscopic examination may also show associated anomalies such as:

- laryngomalacia (5 to 33%) [11,35,37]

- tracheo-bronchial dyskinesia [11]

- tracheo-bronchomalacia, mostly in type III and IV clefts [12,38]

- tracheo-oesophageal fistula [20]

- GERD [11,16,18].

#### Radiological assessment

Routine chest X-rays are usually not conclusive and may only provide signs of parenchyma anomalies secondary to aspiration. It has been reported on CT-scans, in some patients, that an abnormal communication and a lack of soft tissue exists between the trachea and the oesophagus can occasionally be found [39], as well as an abnormal anterior or intra-tracheal position of a nasogastric tube [6,10]. However, neither standard X-ray examinations nor CT-scans are commonly used to diagnose an LC. Similarly, MRI scans are also not commonly used in the diagnosis of LCs, but they are usually needed to assess the associated malformations.

A barium swallow study, most often used to locate a tracheo-oesophageal fistula, will immediately show the flow of the barium into the trachea thereby possibly leading to a false diagnosis of a tracheo oesophageal fistula due to a lack of knowledge of laryngeal clefts by radiologists [15].

#### Diagnostic methods and complemenraty investigations

Once a LC has been diagnosed, a systematic course of action is critical. This includes, in addition to the endoscopic assessment, at least [1]:

- A genetic counselling with family history, full clinical examination and systematic karyotype. According to the clinical findings, specific genetic gene anomalies will be looked for by cytogenetic techniques (e.g. MID-1 for Opitz G, TBX1 for 22q11 microdeletion, GLI3 for Pallister Hall, CHD7 and SEMA3E for CHARGE...). However, in some cases, even presenting with typical clinical features, no specific genetic anomalies can be found.

- Cardiac and renal ultrasonographies

- Spinal (cervical) x-rays

- Hearing screening

In the frequent case of associated malformations requiring surgery, the choice of the surgical sequence will be made according to the child's condition and respiratory status.

### Treatment

#### Medical management

Medical management aims to: 1- maintain satisfactory ventilation in children presenting an obstructive form of LC (mostly by prolapsing mucosa), 2- prevent secondary pulmonary complications as a result of repeated aspiration and 3- ensure adequate feeding of the child.

In cases of respiratory distress (possibly neonatal), an endotracheal intubation may be required. This procedure carries for LCs (except for grades 0 and I), a high risk of tube misplacement and should be undertaken under endoscopic control. For grade 4 LCs, the stability of the tube in the airway and the quality of mechanical ventilation may be difficult to maintain. Placing the tip of the tube very close to the carina is helpful. If a tracheotomy is decided upon, the placement of the cannula also requires an endoscopic control. In extreme type IV LCs, trachea and oesophagus are merged in one single tract and ventilation is extremely hard to maintain; the prognosis is therefore very guarded [6,15,19,39,40].

Noninvasive positive pressure ventilation (continuous or bi-level positive air pressure) is not recommended in children with a non-operated LC, because of: 1- the mobile, mucosal, obstructive component of the airway which can be displaced by the positive pressure, thus worsening the obstruction and 2- the increased risk of secondary pulmonary infection resulting from the aspiration, increased by the air flow. However, in a case of relapse of obstructive symptoms after surgery, this non-invasive ventilation technique may be helpful.

In a recently operated upon child, an endotracheal intubation (e.g. for a secondary respiratory distress) should be approached with extra care, for it can compromise the surgical reconstruction before healing is complete.

Children with a mildly symptomatic type I LC may be fed with thickened food, generally with good success. GERD treatment and postprandial upright position are also helpful [34,41,42]. Patients with a symptomatic type I or II LC will benefit from an anti-reflux treatment and nasogastric tube feeding [29]. In some cases of significant type III or type IV clefts, the risk of aspiration is so high that parenteral nutrition may temporarily be necessary [39,43]. High grade LCs often require a mid- to long- term gastrostomy (often with fundoplication) [11,35,37]. Gastric division with a proximal drainage tube and distal gastrostomy have also been proposed for type IV LCs [36,44-46].

#### Surgical management

Two surgical standards exist for LCs: the external and endoscopic approaches. The classical, systematic, external approach has been partially replaced by endoscopic procedures during the last decade. However, high grade LC or cases of endoscopic failures still require a classical approach.

##### • External approach

###### Approaching the cleft

Depending on the type of LC and the experience of the surgical team, different cervical approaches are possible: lateral with lateral or posterior pharyngotomy, and anterior laryngotracheal.

The lateral approach with lateral pharyngotomy has been recommended for low grade LCs with less than 2 cm of cervical trachea involved [6]. Its drawback is the risk of recurrent and pharyngeal injuries to the nerves The lateral approach with posterior pharyngotomy is seldom used [15]. The risk of nerve injury is lower, but the control of the upper part of the cleft in the interarytenoid region can be difficult [19].

**Figure 4:**
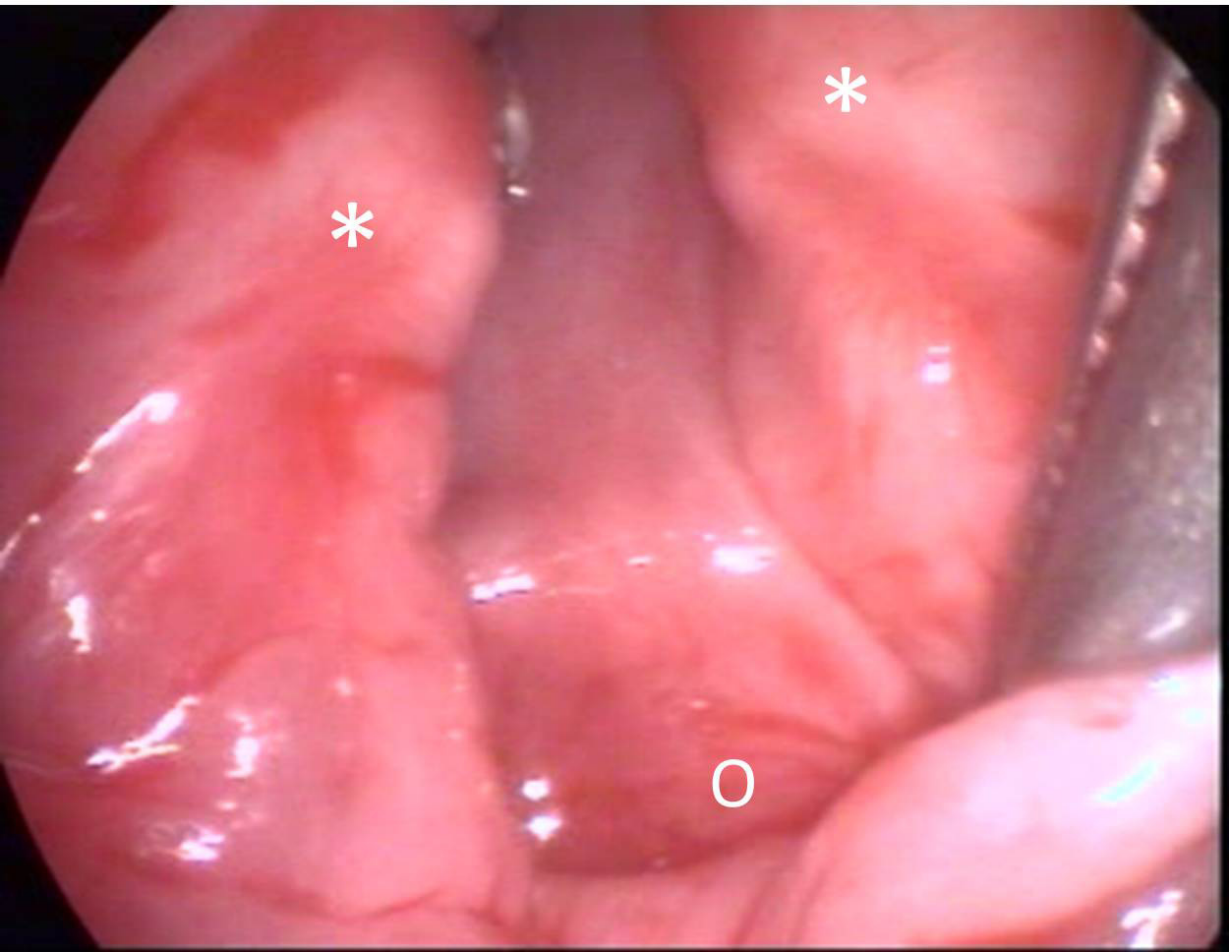
Type III LC, per-operative anterior view (external approach); *: arytenoids; o: esophagus. The posterior wall of the pharynx is visible thru the cleft.

The anterior laryngotracheal approach is the most widely used open technique. It provides an excellent exposure of the cleft with minimal neck dissection, and presents no risk of nerve damage (Figure 4). Once the cleft is closed, it is fundamental to ensure the postoperative stability of the larynx. Despite early concerns, it has been shown that the larynx has a normal growth pattern after such a procedure [47]. In brief, the skin incision is horizontal at the level of the cricothyroid membrane. After sectioning off the thyroid isthmus, the larynx is opened vertically, in accordance with the width of the cleft. The airway can be maintained per-operatively by either a tracheotomy [19,48,49] or an endotracheal tube placed in the tracheal incision and replaced by a standard intubation at the end of the procedure [1,34,47]. Our preference is for the latter technique, which avoids: 1- an additional tracheal trauma, 2- a potential tracheal ischemia by compression, and 3- the morbidity of a paediatric tracheotomy.

Two types of thoracic approaches have been described: anterior (with a sternotomy) and lateral; both combined with a cervical incision [20,44,46,50-53]. Combined cervical and thoracic approaches are only indicated in type IV clefts, and may also require cardiopulmonary bypass or extracorporeal membrane oxygenation [51-53].

#### Closing the cleft

Several closure techniques have been described: 1- a multi-layer closure after excision of excess mucosa [34,54] and 2- the use of asymmetric flaps with non-overlapping suture lines [39,48]. The first concern with both those reconstructive techniques is the risk of ischemic damage on the recently created suture line by the endotracheal tube, a tracheotomy or the nasogastric tube. Because of this risk, the authors believe that the use of a temporary endotracheal tube is preferable to a tracheotomy [34,51]; similarly, a gastrostomy is preferable to a long-term nasogastric feeding tube.

In order to lower the risk of secondary opening, interposition grafts can be used: tibial periosteum or auricular cartilage [34], sternocleidomastoid muscle flap [20], fascia temporalis [55], costal cartilage [56], pleural flap [43,50,57], or even pericardium [51]. The authors prefer tibial periosteum as primary grafting material because it is easy to harvest and manipulate, and due to the satisfying results experienced by the authors in this indication [29,58].

Even after an uneventful procedure, and irrespective of the technique used, the risk of relapse of the LC (secondary re-opening of the cleft) requiring a revision is high: currently documented at 11% to 50% [29,43,46,58-61]. Thus, multiple procedures are common in LC management.

Tracheomalacia is a frequent issue in the post-operative management of type III and type IV clefts, and may require the use of a stent by tracheotomy [45,46,51], excision of the malacic segment [12], aortopexy [12,46], or positive pressure ventilation [34,37,38,45,62].

The latter is a non-invasive technique, which generally allows a satisfactory control of the obstructive symptoms without additional surgical procedures, and, in the authors opinion, should be the intended first.

##### • Endoscopic approach

Since its early beginning in the 1980's, numerous publications have described the endoscopic technique for the closure of type I and type II LC [8,29,41,55,58,60,63,64]. Recently, successful cases of endoscopic closure of type III clefts have also been reported [58,60].

Endoscopic surgery has many advantages: no skin incision, no laryngeal opening, and the possibility of repeating the surgery easily without great additional morbidity. It is performed under general anaesthesia with spontaneous breathing and thus requires an experienced team of paediatric otolaryngologist surgeons and anaesthesiologists. Type I clefts have been closed endoscopically with an endotracheal tube in the airway [6], but spontaneous breathing allows an optimum exposure of the operating field itself.

The cleft closure is performed under suspension laryngoscopy, using a microscope and specific cold instruments: an endoscopic needle driver and a knot pusher [29,58,60]. Most authors also prepare the cleft before closure by denuding the mucosal margins using a CO_2 _or Thulium (Revolix^©^, LISA Laser, Katlenburg-Lindau, Germany) LASER. The dissection of the cleft, necessary prior to the two-layer non-absorbable suture (e.g. Prolene^© ^6/0 dual 9.3 mm needle) requires regular microlaryngoscopy instruments (Collin ORL, Bagneux, France) and mostly experience and patience from the surgeon and his assistant. The sutures are ideally placed on the pharyngeal side of the posterior wall of the larynx, in order to avoid irritation of the larynx and fall into the airway. In neonates or small infants, especially with a respiratory condition, the realization of a 2 layer suture may be difficult to achieve. In these cases, one must be careful to perform at least a 1-layer suture closure with 3 or more sutures. The sutures have to be tight enough to limit secondary opening of the cleft, but must not narrow the laryngeal lumen or be an obstacle to arytenoid movement.

The patients usually spend the first night in intensive care because of the risk of secondary oedema leading to respiratory distress, and can be fed after 7 to 14 days. Some authors prefer a re-intubation for a few days [18], and others non-invasive ventilation [8].

Endoscopic LC surgery has satisfactory success rates: 80% to 100% [1,8,11,18,29,41,58,60].

Endoscopic positioning of an interposition graft is technically very difficult, not to say impossible. However, authors have experienced the use of complementary materials, with varying success rates: collagen [65], Gelfoam^® ^[66], and more recently bioplastic [64], in order to improve long-term results, Bioplastic beingless absorbable than the previous materials. These procedures with injectable materials have been mostly proposed in type I clefts and remain used by few teams. Moreover, in light of the good results of endoscopic procedures without injectable materials, long-term results involving more patients are required before these techniques can be recommended.

The low morbidity and the favourable results of the endoscopic approach have made it the technique of choice for type I and II LCs. Moreover, several cases of type III LC successfully treated by endoscopic means have recently been reported [1,29,58,60]. However, it is likely that despite the advances in surgery and anaesthesiology, some type III and all the type IV will never benefit from the endoscopic approach.

##### • Therapeutic timing and strategy

The prognosis of LC is closely linked to the grade of the cleft, the associated conditions, and the pulmonary status of the patient. In all cases, surgery should be performed as soon as possible to avoid complications related to the aspiration and gastric reflux [11,29,41,51]. However, the degree of severity and thus the need for rapid management is very different between the different forms of the disease.

Submucosal clefts can be especially problematic. In the rare cases where they are the only malformation and have no clinical impact, a watchful surveillance can be proposed. However, the presence of a submucosal cleft has been described as a contributing factor to the failure of an airway reconstruction procedure. Thus, this type of cleft should be taken care of concomitantly with to the other malformation, usually with a posterior graft via an external approach [30].

A toddler with a grade 1 LC presenting with mild symptoms and occasional aspiration - without associated conditions - will be managed very differently to a newborn with a grade 4 LC, (massive saliva aspiration, unstable endotracheal intubation, associated great vessel malformations) and whose survival will be compromised during the first hours of life.

### Prognosis

During the recent decades, advances in both medical care and surgical techniques have notably improved the prognosis of LCs. The overall mortality rate in a series of patients in 1983 was 46% [4] and has dropped to 6% - 25% in most recent reports [54,55,58,60]. This improvement in survival can be explained not only by the advances in treatment and management, but also by an earlier diagnosis. Indeed, the sooner a diagnosis is made, the greater the reduction in morbidity and mortality.

However, comparing different series is difficult because of: 1- the low incidence of pathology, 2- the inhomogeneity of the groups of patients in these series, 3- the important differences in clinical presentation and prognosis of the different grades of LC, and 4- the impact of associated malformations. The mortality rate for type IV LC only, for example, was estimated at 93% in 1985 [4] and is now below 50% [32,37,54].

## Conclusion

LCs are rare malformations of the larynx, whose prognosis is highly dependant on the extension of the cleft and other associated malformations. The origin and development of this malformation is as yet not perfectly understood. Isolated LCs are unusual but possible. The management of low-grade LCs (I, II, and some III) has recently been drastically improved by the development of endoscopic surgical techniques, leading to significant improvements in survival and quality of life of patients. However, high-grade LCs or recurrences will still necessitate an open approach procedure.

## List of abbreviations

LC: laryngo-tracheo-oesophageal cleft.

## Competing interests

The authors declare that they have no competing interests.

## Authors' contributions

Both authors read and approved the final manuscript.

## Author's information

The work of NL is supported by the *Société Française d'ORL *(SFORL).
